# Supplementary material for: The True Nature of Tricalcium Phosphate Used as Food Additive (E341(iii))
Source: Nanomaterials (Basel). 2023 Jun 8;13(12):1823. doi: 10.3390/nano13121823 (PMC10303396; doi:10.3390/nano13121823)
Supplement: Supplementary file 1 [file nanomaterials-13-01823-s001.zip › nanomaterials-2407627-supplementary.pdf]

Application of the Rietveld method to quantify potential amorphous phase in E341iii A

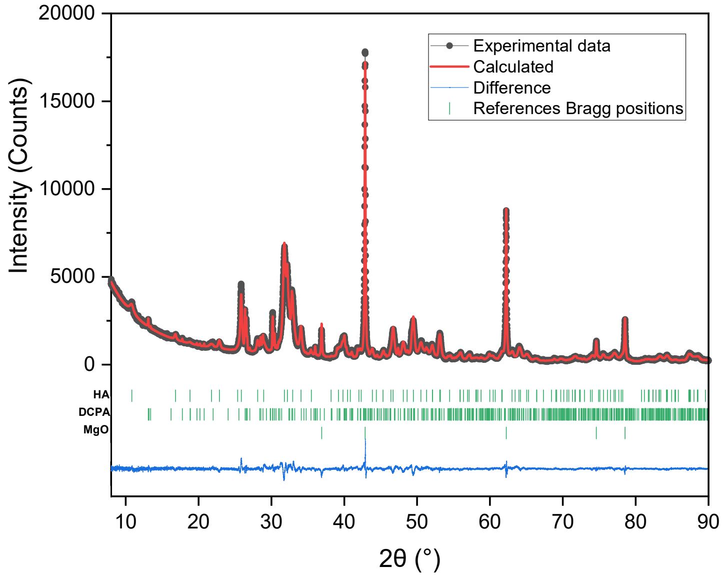

Figure S1. Experimental, calculated and difference X-ray diffraction pattern of E341iii A including a known proportion of MgO. The calculated amount of MgO matched the one introduced revealing the absence of amorphous phase in the sample.

Comparison of dissolution rate of E341iii A and DCPA

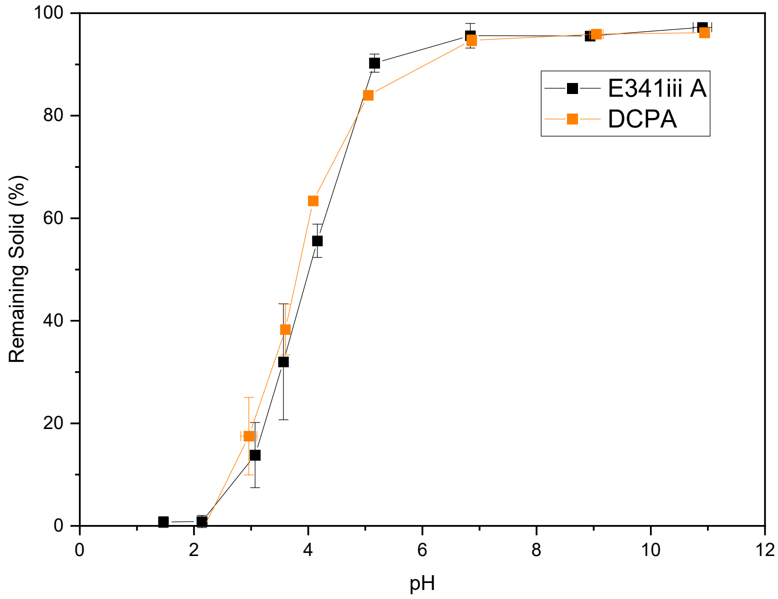

Figure S2. Remaining solid after placing 100 mg (initial mass) of E341iii A and DCPA (obtained from the same manufacturer as E341iii B) in 20 mL of ultrapure water at different pH values for 48h at room temperature with moderate stirring (40rpm).

Sedimentation rate

The sedimentation rate was determined using a Perkin Elmer lambda 1050 UV-Vis spectrophotometer. The measurement was performed at 230 nm every 5 seconds. The prepared 0.1% (w/v) E341iii aqueous solution was manually agitated in a quartz cuvette before the beginning of the measurement.

As observed by TEM and granulometry, bare HA nanoparticles tend to form agglomerates when placed in an aqueous medium. These agglomerates do not form stable suspensions and their sedimentation rate is of importance when discussing the physico-chemical properties. The presence of salts or proteins can also influence suspension stability [33]. The sedimentation of the three E341iii samples is relatively fast but shows noticeable differences (Figure 13). The wavelength used to follow sedimentation (230 nm) is not typical but presented better results than typical wavelength (unsuccessful attempts at 850 nm and 900 nm). The optical properties of the studied samples, presenting a low absorption in the visible range, did not allow for precise sedimentation monitoring at usual wavelengths for such determination.

The sedimentation half-times of E341iii A, B and C are respectively of 32 min, 26 min and 41 min. These results can also be expressed as the time when all the particles and agglomerates alike have settled and no significant variation of A/A_0 is observed. Hence, in the case of E341iii B, sedimentation is over after 1.8 h while E341iii A requires 2.4 h and E341iii C 3.7 h.

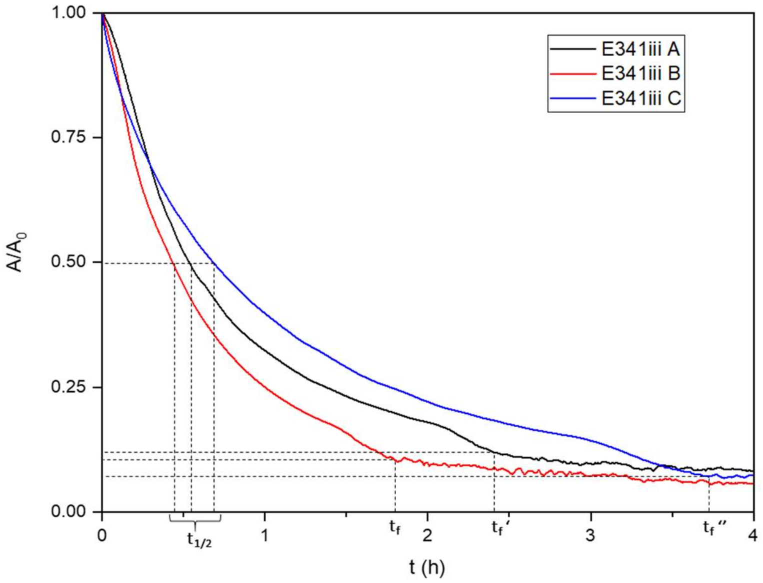

Figure S3. Normalized absorbance (at 230 nm) of E341iii A, B and C (pH respectively of 6.67, 6.96 and 7.23) as a function of time showing the progressive sedimentation of particles in water. Half-times ($t_{1/2}$) and final sedimentation (t_f) are indicated.

Overview of the particle dispersion attempts methods

Table S1 – Summary of some of the methods used to attempt to separate nanoparticles (here for E341iii B – and one example with E341iii C).

**Sample E341iii B** – Type of Method	Image example	Comments
**Chemical** functionalization attempt with Triméthyl(tétra-décyl)ammonium bromide (0.01 M) in water	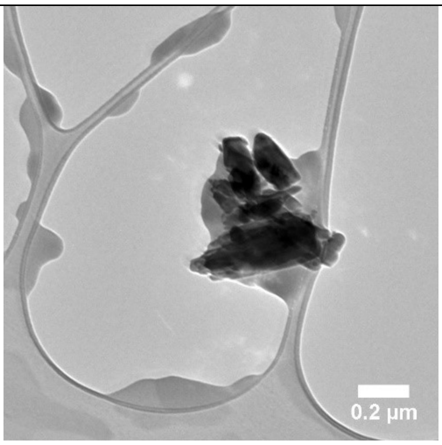 HNAR 9000	-No significant particle separation improvement
**Chemical** functionalization attempt with lysozyme (1 g/L) at pH 9	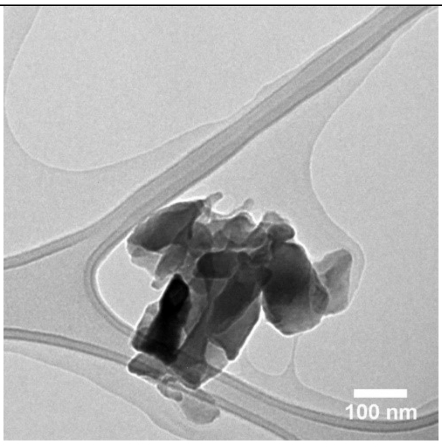 HNAR 9000	-No significant particle separation improvement
**Physico-chemical** method combining the functionalization attempt with lysozyme (1 g/L) at pH 9 and sonication (40 %, 5 min)	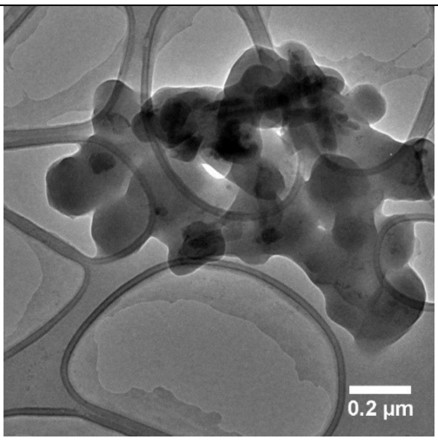 HNAR 9000	-No significant particle separation improvement<br>-Visible particle degradation

**Chemical** functionalization attempt with BSA (1.5 g/L)	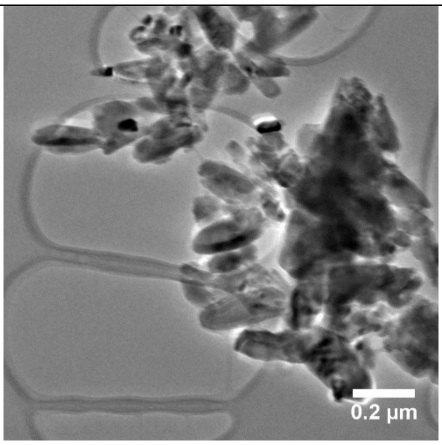<br>HNAR 9000	-No significant particle separation improvement
**Physical** separation attempt by sonication (80 %, 10 min) in water	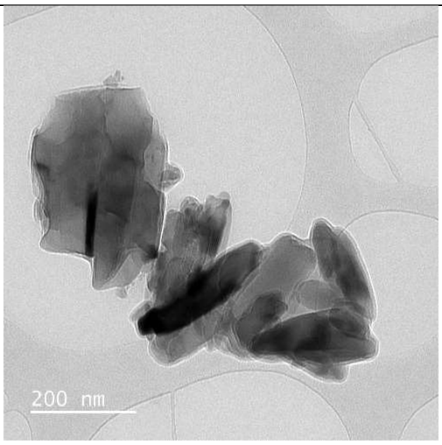<br>Themis Z G3	-No significant particle separation improvement<br>-Some images seemingly show partial particle degradation/alteration
**Physical** separation attempt by sonication (40 %, 5 min) in ethanol	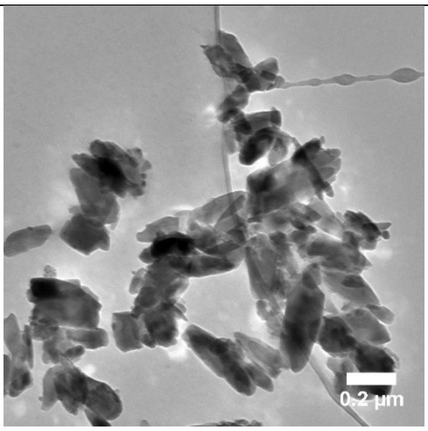<br>HNAR 9000	-No significant particle separation improvement<br>-Some images show partial particle degradation/alteration
**Physical** separation attempt by tape method	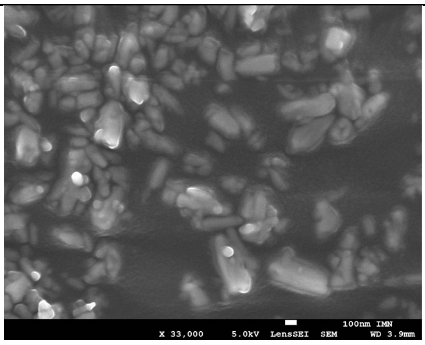<br>JEOL 7600F	- Clear particle separation<br>- Reproducibility needs improvement<br>- Lack of image resolution as SEM apparatus is less performant than TEM (hence the transfer attempt from tape to TEM grid)<br>-possible adhesive overlapping particles making their edges less clear

E341iii B – **Physical** separation attempt by spin coating (1500 tr/min)	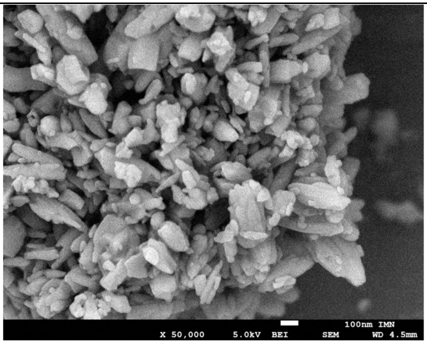 JOEL 7600F	-No significant particle separation improvement
E341iii B – **Physical** separation attempt by high pressure homogenizer (85 bars)	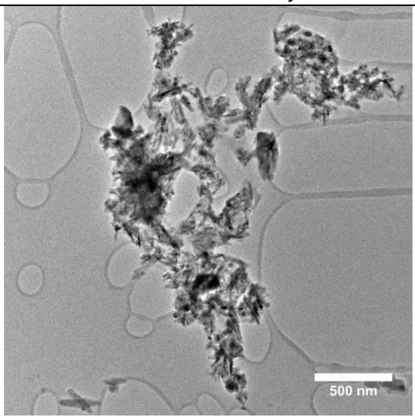 Themis Z G3	-No significant particle separation improvement<br>-Visible particle alteration
**Physical** separation attempt by tape method (transfer onto TEM grid by dissolving tape with acetone) **E341iii C**	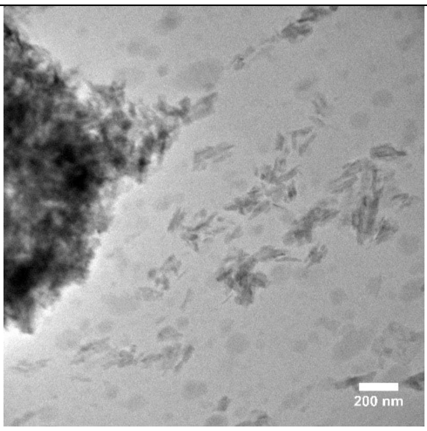 Themis Z G3	-Some particle separation observed (few usable areas)<br>-Method needs improvement as the transfer is rendered difficult by the tape removal from the grid (tape dissolution with acetone left important amount of material blocking large areas of the grid)

The TEM images were captured either on a S/TEM Themis Z G3 (Thermo Fischer Scientific) operating or a HNAR 9000 (Hitachi) operating at 300kV. The SEM images were captured on a JEOL JSM 7600F microscope. Typically, a few milligrams of sample were placed in 20 mL of solvent before the deposition on a lacey or holey carbon covered copper grid (for TEM samples).

Tape method consisted in the successive transfer (usually 5) of a powdered covered tape (a few milligrams were deposited on tape) to a new piece of tape by sticking both pieces together. A transfer was completed upon unsticking the tape pieces. The newly sample covered tape is used. After sufficient cycles, the tape is cut and placed on SEM stub where it is coated with platinum and analyzed.

Dispersion through sonication was performed using a cup horn sonicator (20 kHz, 500 W) for a sample volume of 20 mL. TEM samples were prepared immediately after sonication.

33. Tercinier, L.; Ye, A.; Anema, S.; Singh, A.; Singh, H. Adsorption of Milk Proteins on to Calcium Phosphate Particles. *Journal of Colloid and Interface Science* **2013**, *394*, 458–466, doi:10.1016/j.jcis.2012.11.058.
